# Meta-analysis of *H. pylori* and the gut microbiome interactions and clinical outcomes

**DOI:** 10.3389/fcimb.2025.1610523

**Published:** 2025-08-01

**Authors:** Xiongjian Wu, Haiyan Zhu, Ying Hu, Lei Zhang, Lixing Huang

**Affiliations:** Department of Gastroenterology, the First Affiliated Hospital of Gannan Medical University, Ganzhou, Jiangxi, China

**Keywords:** meta-analysis (MA), *H.pylori*, gut microbiome (GM), predictive model (PM), clinical applications

## Abstract

**Introduction:**

*Helicobacter pylori* is a globally prevalent gastric pathogen associated with chronic gastritis, peptic ulcers, and gastric cancer. Its interaction with the gut microbiome (GM), a dynamic microbial community within the gastrointestinal tract, plays a critical role in modulating host immune responses and disease progression. This study aimed to investigate the complex interactions between *H. pylori* infection and the GM and to evaluate how microbiome alterations relate to clinical outcomes such as gastritis, ulcers, and gastric cancer.

**Methods:**

A meta-analysis was conducted using publicly available 16S rRNA and shotgun metagenomic datasets. Microbiome composition differences were assessed using differential abundance analysis, alpha- and beta-diversity metrics, and principal component analysis (PCA). Random forest models were employed to predict the clinical outcomes based on microbiome and clinical data. Hyperparameter tuning and cross-validation were applied to ensure model robustness.

**Results:**

The analysis revealed significant microbial shifts associated with *H. pylori* infection, including enrichment of *Proteobacteria, Fusobacterium* spp., and *Prevotella* spp., and depletion of beneficial taxa like *Lactobacillus* spp. and *Faecalibacterium prausnitzii*. Microbial diversity declined progressively with disease severity. Predictive models demonstrated high accuracy (89.3%) in classifying the disease states and identifying key microbial biomarkers such as *Fusobacterium* spp. and *Bacteroides fragilis* with strong predictive power.

**Discussion:**

This study highlights the critical role of GM dysbiosis in *H. pylori*-related disease progression. The identified microbial signatures and predictive models offer promising tools for early diagnosis, risk stratification, and personalized treatment of *H. pylori*-associated gastrointestinal disorders. Future integration of multi-omics data may further unravel the microbial mechanisms and support microbiome-based precision medicine.

## 1 Introduction

*Helicobacter pylori*, a Gram-negative bacterium, is widely acknowledged as the primary etiological agent of chronic gastritis and peptic ulcers, and is a significant risk factor for gastric cancer (GC) (Suerbaum & Michetti, 2002). This pathogen infects approximately half of the global population, with a particularly high prevalence in developing regions, and has been linked to a range of gastric pathologies that vary in severity from benign gastritis to life-threatening cancer (Yamaoka and Graham, 2014). The persistent infection with *H. pylori* is complex, influenced by both host factors and environmental conditions, and the presence of a diverse gut microbiome (GM) has emerged as a significant modulator of the infection’s progression and outcomes (Fakharian et al., 2022). The GM is a diverse and complex community of microbes, residing in the human gastrointestinal tract, and is now more recognized as an essential element that influences the host’s immune responses, metabolic activity, and disease development (Rinninella et al., 2019).

In infected individuals, analyzing the changes in microbial composition that relate to clinical outcomes is crucial, because the relationships between *H. pylori* and the microbiome is complex (Benghezal et al., 2014). This analysis thus helps in improving diagnostic plans. The severity and outcomes of *H. pylori* (Xie et al., 2024)*-*related diseases are greatly impacted by the GM, and it also impacts the modulation of immune response, as demonstrated by a recent study (Bolyen et al., 2019). Some microbiome profiles could aggravate or reduce the impact of *H. pylori* infection on clinical symptoms including gastritis, ulcers, and even stomach cancer (Gorkiewicz and Moschen, 2018). Many studies have revealed that the variation of GM diversity and the abundance of particular microbial groups differ greatly between (Dash et al., 2019) *H. pylori*-infected people and those who are healthy, suggesting a notable interaction between microbial populations and host disease outcomes (Mira-Pascual et al., 2020).

Our understanding of these interactions remains poor despite these results since the great diversity among many populations and the difficulty in conducting large, long-term clinical studies limit our knowledge (La Placa et al., 2025). Despite having certain restrictions like small sample sizes, population diversity, and the long-term characteristics of many disease outcomes, conventional experimental studies and clinical trials have been instrumental in improving our knowledge of the interactions between *H. pylori* and the microbiome (Yang et al., 2019). Detecting consistent microbiological fingerprints over different populations is complicated, because of the wide range of clinical presentations linked to *H. pylori*-related diseases (Zhang et al., 2021). Thus, a comprehensive technique is needed because of this complexity. This comprehensive technique fuses the outcomes of various studies. An applicable solution to complexities is attained by the statistical method named meta-analysis (MA), as it integrates data from various studies.

In a single study, vital patterns and trends are unnoticed, because of the small sample size or methodological differences. However, outcomes of various studies in MA may detect vital patterns and trends effectively (Borenstein et al., 2009). The precision and reliability regarding the relationship between the GM and the *H. pylori* disease were improved by MA, as it enables to evaluate applicability of outcomes across different demographics, experimental settings, and geographic areas (Suerbaum & Josenhans, 2007). *In silico* methods, especially MA, become more crucial in microbiome studies. This MA supports extracting important insights from large publicly available datasets. The analysis of complex relationships among microbial populations, host characteristics, and disease outcomes is made possible by MA, as it integrates several microbiome data from various studies using computational techniques. This is effective in analyzing *H. pylori* infection (Rinninella et al., 2023).

Large-scale, multi-cohort studies are becoming difficult because of the severity of the disease and clinical variations among infected individuals (Agamah et al., 2020). The hidden patterns in microbiome data are identified by *in silico* methods, as they integrate the data and promote the application of advanced statistical and machine learning (ML) models (Aparicio et al., 2020). *In silico* methods also offer more comprehensive perspectives regarding how microbial processes function in terms of controlling disease progression. To analyze the relationships among *H. pylori* infection, the GM, and related clinical outcomes, MA and *in silico* methods are employed in the current study. There are three main objectives in this study: (1) the microbiome data from public datasets are integrated and contrasted; (2) modifications in the microbiome and its relationship with clinical outcomes including gastritis, ulcers, and GC are studied; and (3) the disease outcomes based on microbiome characteristics are predicted by the creation of predictive models (PMs).

The primary objective is to methodically gather and integrate data from prior studies that investigated the microbiome with *H. pylori* infection utilizing modern sequencing technologies including 16S rRNA gene sequencing and shotgun metagenomics (Benghezal et al., 2014). The GM over several populations and clinical outcomes is consistently detected by the MA method. MA helps in the detection of the vital microbial markers related to disease progression. Analyzing how GM changes caused by *H. pylori* infection are strongly correlated with clinical outcomes is considered to be a secondary objective of this study. Clinical outcomes from multiple studies, combined with microbiome data, enable the identification of microbial signatures that are strongly associated with diseases such as gastritis, peptic ulcers, and gastric cancer. Recent studies have underlined the importance of specific microbial taxa in changing the immune responses and influencing inflammatory mechanisms linked to these conditions; this MA seeks to identify the consistent biomarkers for disease severity and progression across several cohorts (Mira-Pascual et al., 2020).

The final goal of this study is to implement predictive modeling to assess how the status of *H. pylori* infection, together with microbiome data, could forecast clinical outcomes. The study aims to find significant microbiological traits and clinical variables that can predict disease outcomes by using ML methods on the combined dataset. For personalized medicine, this PM may be crucial since it would allow doctors to use the microbiome data to guide treatment decisions, hence improving patient outcomes by more tailored therapy strategies.

This work aims to provide a complete knowledge of microbial pathways contributing to *H. pylori*–related disorders by combining MA of microbiome datasets with computational techniques. Research findings could not only help define new diagnostic techniques but also help provide tailored therapies considering the personal microbiome profiles of *H. pylori*-infected individuals. The combination of MA and advanced computational methods will definitely be vital in elucidating the complex relationships between the human microbiome, pathogens like *H. pylori*, and clinical illness outcomes as studies in the microbiome field continue to advance.

## 2 Materials and methods

### 2.1 Data collection and integration

Publicly available datasets were systematically collected from recognized and well-established repositories including the National Center for Biotechnology Information (NCBI) as well as other credible databases, including Web of Science and Scopus, to conduct in-depth MA of the GM in *H. pylori* infections. This MA focused on merging the data from studies using 16S rRNA gene sequencing and shotgun metagenomic sequencing techniques, which are vital for providing both wide-ranging taxonomic profiles and detailed functional insights into microbial communities in the human gastrointestinal tract. A comprehensive literature search across four major scientific databases—PubMed, Scopus, Web of Science, and Embase—is conducted. The search aimed to identify studies investigating the interactions between *H. pylori* infection, the GM, and related clinical outcomes in human populations.

Sequencing methods are beneficial for detecting microbial changes. Such methods offer a better understanding of the potential of microbiomes related to *H. pylori* infection (Li et al., 2023). Clinical metadata specially focused on the disease condition of *H. pylori* and related disease concerns, including chronic gastritis, peptic ulcers, and stomach cancer; thus, clinical metadata were collected along with sequencing data. These clinical data are required to link modifications in microbial composition and disease features. A complete picture regarding the *H. pylori* interacts with the GM in influencing the disease progression also offered by these clinical data. The impact of the GM on immune responses and the severity of *H. pylori*-related disorders are highlighted in a recent MA (Fakharian et al., 2022).

The application of strict inclusion criteria (SIC) helps maintain the reliability of merged datasets. The microbiome data reflecting the specific physiological condition of disease related to *H. pylori* are ensured by this method. From a valid study, microbiome sequencing data and comprehensive clinical data regarding the outcomes of diseases including gastritis, ulcers, and stomach cancer are also ensured. These selection criteria thus facilitate a direct correlation among the changes in microbiome composition and clinical outcomes of *H. pylori* infection. The accuracy of this study is improved by the MA method, as it integrates the data over several datasets (Huang et al., 2023).

For valid dataset comparisons, a universal reference framework is created for the standardization of microbial taxonomy data from metagenomics and 16S rRNA sequencing investigations, and it is considered to be the second crucial element of this study. This study also ensures the consistency and reliability of the outcomes over studies. Taxonomic data from established microbiological databases were compared to ensure that microbial taxa were consistent across studies. A consistent framework was also employed for organizing these clinical data. *H. pylori* infection status, disease stages, patient demographics, and treatment histories are all included in the clinical data. The high-quality, well-annotated dataset is created by this methodical process, and it will help in analyzing the complex relationships between the GM and the issues related to *H. pylori* infection (Sharma et al., 2021).

This integrated dataset provides a detailed comparative analysis regarding the microbiome compositions across a number of clinical outcomes associated with *H. pylori* infection. Then, the application of the MA and advanced computational methods may assist in the detection of microbiological signs associated with disease progression that includes the modification from gastritis to ulcers and GC. For *H. pylori*-related disorders, these microbial biomarkers contribute to early diagnostic tools, prognostic indicators, or treatment targets. Here, customized treatment plans for the *H. pylori* infection are crucial, based on recent advancements in microbiome studies, and the PM is needed (Zhang et al., 2021).

**Figure 1:**
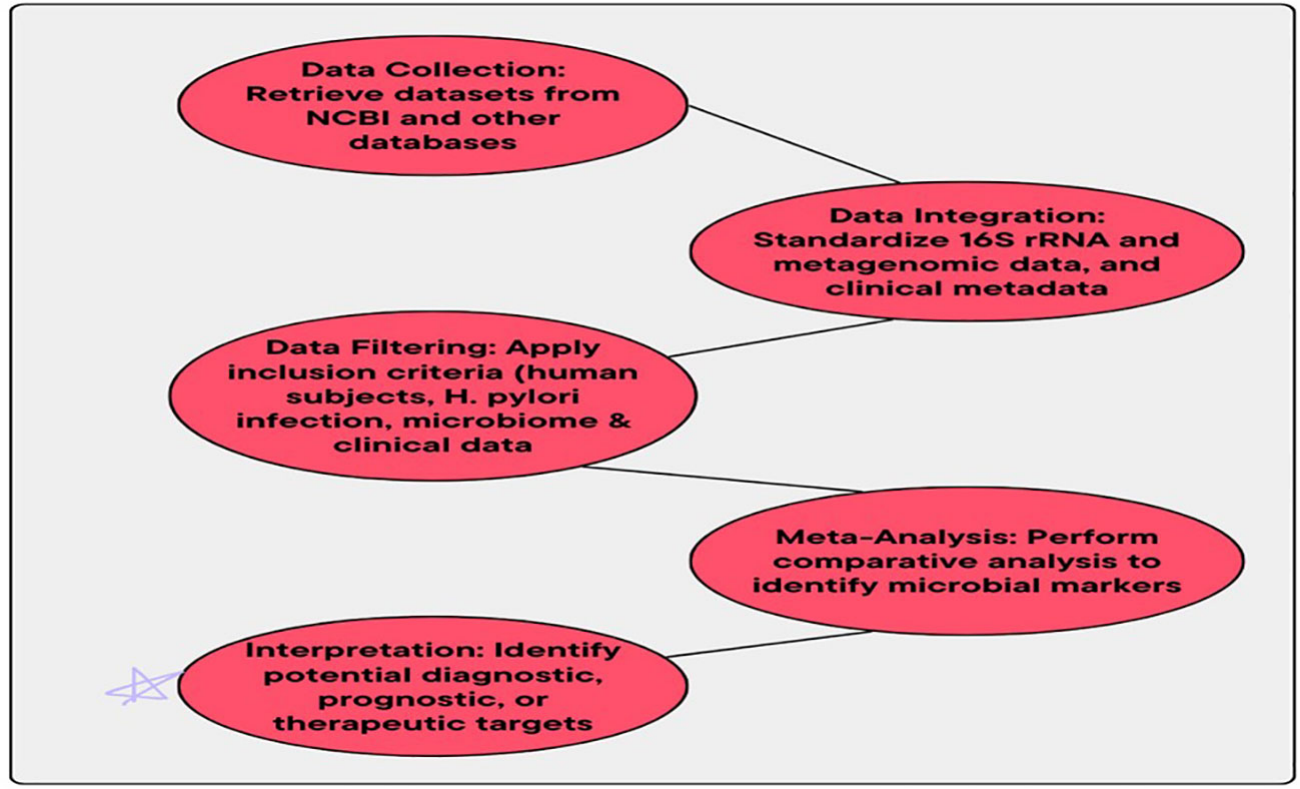
Workflow for data collection and integration.

For future studies, this systematic and comprehensive method to data collection and data integration develops a strong basis. Then, the complex relationship between the GM and *H. pylori* infection is effectively extracted by this strong dataset. The MA method offers a deeper understanding of the way the microbiome alterations impact the disease outcomes. This is done by MA, as it integrates the data from multiple datasets, making this MA method effective. Diseases related to *H. pylori* need an effective and customized therapy, so for this purpose, this application also facilitates in providing a customized therapy. Workflow for data collection and integration is presented in **Figure 1**.

### 2.2 Comparative analysis of microbiome composition

The distinctive microbiome signatures are identified, and it is highlighted in the study. By using these distinctive microbiome signatures, the GM compositions between healthy subjects and *H. pylori*-infected subjects are contrasted in this study. Here, the microbiome changes related to *H. pylori* infection are associated with several clinical outcomes; in this case, one may understand that it is vital to be aware of the role of pathogens in gastrointestinal disease (Zhang et al., 2021; Li et al., 2023). With the support of reliable tools like DESeq2 (Love et al., 2014) and edgeR (Robinson et al., 2010), microbial data were effectively identified by the differential abundance (DA) testing. When comparing healthy controls (HC) to people with *H. pylori* infection, these microbial taxa are more or less prevalent. In microbiome studies, these techniques are often utilized. Then, detecting taxa is also facilitated by this method. The study highlights differences in microbial taxa when compared to control groups. Certain microbial taxa were found to have distinct patterns in abundance across different groups. The research emphasizes the statistical significance of these differences in microbial presence. Overall, the findings suggest that microbial diversity varies significantly between the studied groups and the controls (Wang et al., 2020).

Then, a novel statistical method—the Benjamini–Hochberg (BH) procedure—is employed by researchers. The BH procedure is employed for the purpose of minimizing the probability of the false detection rate (FDR). The complex microbiome data are studied via the application of this procedure. In several datasets, the FDR is effectively minimized by this method (Bogomolov et al., 2021). Finally, it also helps in minimizing the Type I errors in case of large-scale analysis. The reliability of the outcomes is ensured by this BH procedure. Generalizability of the findings is evaluated by this MA approach since it pools the microbiome findings together across studies. Such pooling is the main strength of this MA approach. Findings from various cohorts, study designs, and sequencing platforms are pooled together by this MA approach, making it cost-effective. There are built-in variabilities among the microbiome studies (Zhang et al., 2022).

A more comprehensive and integrated comparison of microbial structure between infected and healthy groups is offered by this MA strategy, because it aggregates the results of several studies. The microbiome study is employed for identifying issues regarding the heterogeneity of data. Once the issues are addressed, the results are then enriched by this study (Xu et al., 2023). Here, the consistent microbial changes related to the condition of the disease in several datasets are effectively identified by this MA method. The external validity of the outcomes is also improved. In the *H. pylori*-infected group and HC group, one can determine the variations of those in microbial structure; beta-diversity analysis is used. Then, the outcomes are then visualized via the principal coordinates analysis (PCoA)-based Bray–Curtis dissimilarity and UniFrac distances. Here, sample dissimilarities based on the Bray–Curtis dissimilarity and UniFrac distances are estimated by the PCoA.

The presence or absence of taxa is considered by the Bray–Curtis dissimilarity, as it measures the microbial community composition. On the other hand, UniFrac distances consider the evolutionary relationships among taxa. These UniFrac distances are capable of assessing the relatedness of microbial communities. The abovementioned distance measures are employed by microbiome studies for the purpose of determining the clustering patterns that are related to particular disease conditions (Lozupone et al., 2011; Callahan et al., 2016).

Possible clustering patterns in *H. pylori* infection and its clinical conditions such as gastritis, peptic ulcers, and GC are defined by this beta-diversity. Scientists are also able to determine whether the changes observed in the microbiome, which take place during the course of *H. pylori* infection, were common across various populations and geographical locations, using this MA approach. The approach pools together data from various studies. Such consolidation gives rich information regarding how the virus remolds the microbiome and thereby influences the disease outcome (Sharma et al., 2021). Trends of various datasets are merged, and scientists are meant to identify universal microbial biomarkers. Such biomarkers may serve as diagnostic or predictive agents against *H. pylori*-related diseases.

**Figure 2:**
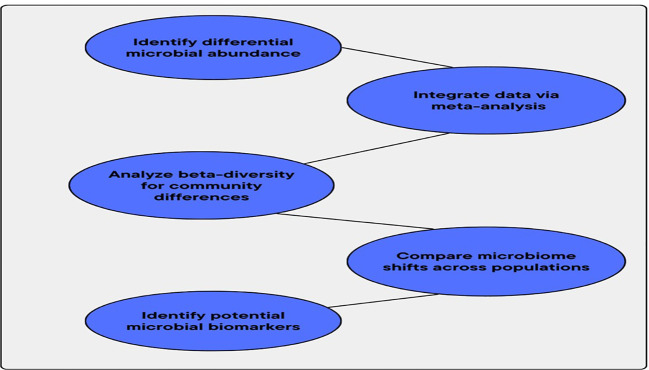
Workflow for comparative analysis of microbiome composition.

A notable microbiome change linked to *H. pylori* infection was identified by beta-diversity studies and differential abundance tests. The integration of several datasets in MA thus enhances the statistical validity of the outcomes. This integration also offers a deeper understanding of the microbiological pathways behind *H. pylori*-related diseases. These outcomes become the basis for detecting microbial biomarkers. Such biomarkers also guide researchers in the creation of focused treatment approaches and accurate therapy plans for individuals with *H. pylori*-induced gastrointestinal diseases. **Figure 2** illustrates the workflow for comparative analysis of microbiome composition.

### 2.3 Association between microbiome shifts and clinical outcomes

The relationships between changes in the GM and clinical outcomes related to *H. pylori* infection are identified by a comprehensive *in silico* MA. To determine both taxonomic and functional features, high-throughput sequencing-based microbiome data, such as 16S rRNA gene sequencing and shotgun metagenomics, were analyzed using QIIME2. The integration of multiple datasets in MA offers a comprehensive insight into how microbial changes in various clinical environments are engaged. Improved classification of *H. pylori*-induced dysbiosis is also facilitated by MA. Studies investigate how microbiome alterations affect disease progression and how clinical conditions are facilitated by the integration of taxonomic and functional profiles with clinical data including gastritis, ulcers, and stomach cancer (Zhang et al., 2023; Li et al., 2022). This MA effectively identifies differences in sample sizes, study designs, and patient cohorts that can potentially make correct associations between the microbiome and the disease. This method offers more generalized, correct insights into microbial compositions involved in *H. pylori* infections, as well as their impact on gastrointestinal disease symptoms. Statistical accuracy is also obtained by this method.

The determination of microbial taxa that are highly correlated to the various clinical outcomes is achieved by differential abundance analysis with the help of the DESeq2 program (Love et al., 2014). The major changes of the gastrointestinal microbiota are due to *H. pylori* infection. Variations in the microbial composition are detected by using these statistical methods. The particular taxa that were consistently elevated or depleted in individuals with gastritis, ulcers, and GC were effectively detected with the help of MA. This will also offer a potential microbiological marker for disease diagnosis and development (Sharma et al., 2021). The outcomes are consistent and pertinent over several populations and therapeutic settings, ensured by the differential abundance testing across many datasets.

The microbial diversity in every disease group is assessed using alpha-diversity measures. The Shannon diversity and observed species are included in the measurements. The abundance and stability of the microbiome in disease outcome are assessed with the help of these measures (Jiang et al., 2024). Pattern identification is considered to be the primary objective of the study. Comparing the diversity between *H. pylori*-infected individuals and different disease conditions makes the pattern identification capable of forecasting the disease process and uncovering early indicators of the disease. MA methods enable the integration of diversity measures across multiple studies; thus, it is essential in this case. A holistic illustration in terms of how the diversity of the microbiome is related to clinical outcomes and disease severity is also provided by this MA method (Liu et al., 2022).

Microbiological determinants that correlate with clinical outcomes are assessed through random forest (RF) classification, a type of ML methodology. Microbial biomarkers were identified utilizing RF classification. The taxonomic data and clinical features were analyzed, thus complementing RF in the detection of microbial biomarkers that correlate with the clinical outcomes. The microbial biomarkers are capable of making predictions by combining with the clinical characteristics of patient demographics and stages of disease. The RF models were successful with high-dimensional and complex datasets common in microbiome studies, and they were able to manage numerous variables in an attempt to establish complex and nonlinear (NL) relationships among microbial taxa and clinical conditions (Ghosh et al., 2021). The RF model can discern significant microbial signatures. These microbial markers may function as predictive markers of disease development and treatment responses.

These principal component analyses (PCAs) are employed for pattern analysis of microbiome composition related to different disease states. In dimensionality reduction (DR), this PCA is mainly employed. The correlations among various samples were determined on the basis of microbiome features by the help of PCA. These patterns of clustering are identified through this method. This clustering technique also identifies the specific disease phenotypes of gastritis, ulcers, or stomach cancer (Wang et al., 2020). The different microbiome profiles associated with each clinical outcome here were identified through the application of PCA in the combined data. The disease microbiological structures associated with *H. pylori* are explained through this application.

**Figure 3:**
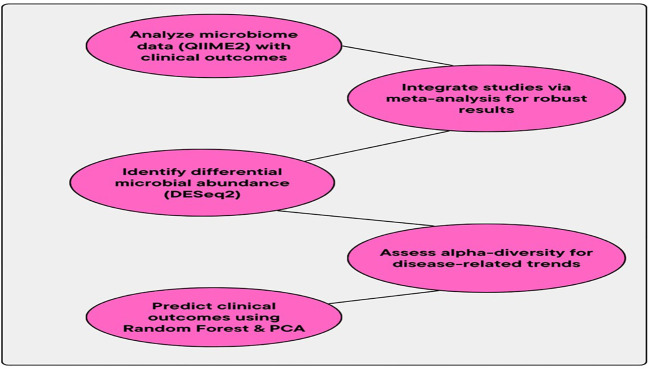
Workflow for association between microbiome shifts and clinical outcomes.

The reliability and practicability of models are ensured by cross-validation (CV) techniques. These CV methods are usually employed by the PM. The overfitting issue is prevented by the CV method. Then, the outcomes are not limited to a specific group of data, also ensured by this CV method (He et al., 2023). The accuracy (ACC), precision (P), and recall (R) of the models are validated by this CV method. The reliability and stability of the identified microbial biomarkers across several datasets are also validated by this CV method. The MA method integrates high-throughput sequencing data with clinical information and advanced computational techniques, and it may offer some crucial insights into the evolving relationship between the GM and clinical outcomes in *H. pylori-*infected individuals. The consistent biomarkers and disease-related patterns are detected by integrating microbiome data from several studies. The creation of diagnostic tools, prognostic models, and individualized therapy regimens for diseases related to *H. pylori* is impacted by these consistent biomarkers and disease-related patterns. The workflow for the association between microbiome shifts and clinical outcomes is provided in **Figure 3**.

### 2.4 Predictive modeling of clinical outcomes

Creating the PM is considered as the third objective of this study, as these PMs have the potential in predicting clinical outcomes based on *H. pylori* infection conditions and related microbiome data. Managing high-dimensional and complicated microbiome data are done via the RF model; thus, it is chosen. The clinical factors including patient demographics (age and gender), infection status, disease progression, and treatment history are included in these data. A type of ensemble learning (EL)—the RF model—is chosen. In microbiome studies, the RF model integrates the predictions from many decision trees (DTs). For handling high-dimensional data, the RF model is effective. The RF model plays a great role in microbiome studies. The RF model may help prevent overfitting issues and also improve the robustness of the model (Liaw & Wiener, 2002).

The RF models are also effective in detecting complex and NL interactions among microbial compositions and clinical outcomes. The RF method is well-suited and effective in this microbiome study (Chng et al., 2021). The MA also offers more comprehensible and generalizable models. The MA method has the potential to create more comprehensive models, as it integrates data from multiple datasets. Here, data are integrated from several clinical cohorts and geographic areas. It enables researchers to maximize sample size and also considers the variability of the study design. This integration will lead to an increase in the predictive value of the model with respect to clinical outcomes (Gao et al., 2023).

Population variability is effectively identified by the MA method, making it crucial, because it is usually limited by smaller sample sizes or population-specific biases. This MA also helps enhance the performance of the PM (Xu et al., 2022). The generalizability of the model is improved by integrating data from several cohorts. This integration will also help identify consistent microbiological markers. These identified biological markers predict disease outcomes over various patient populations. The validity of the model is improved by the cross-validation (CV) method. This CV method splits the dataset into training and validation subsets. This split enables researchers to test and train the model iteratively. This assessment will help improve the generalizability and reliability of the model. This CV method also lowers the overfitting issues.

The performance on novel data was also ensured by CV (He et al., 2023). The performance of the models is evaluated effectively via the support of performance metrics, namely, ACC, P, R, and the area under the receiver operating characteristic curve (AUC-ROC). The prediction ability of the model is evaluated by using these metrics (Yuan et al., 2020). In case of imbalanced datasets, for assessing the balance among the true positive (TP) rate and false positive (FP) rates, this AUC-ROC metric is crucial. PM effectively detects the microbiological structures that are related to clinical outcomes like ulcers, gastritis, or GC. From the matching outcomes of other major MA studies on *H. pylori* infection, it is clear that some taxa like Firmicutes and Bacteroidetes were shown to be increased or depleted in those with severe disease stages (Sharma et al., 2021).

All meta-analyses were conducted using both fixed-effects and random-effects models. Random-effects models were selected as the primary analysis approach due to the anticipated clinical and methodological heterogeneity across studies. Heterogeneity was quantified using the *I*² statistic, with thresholds defined as follows: low (0%–25%), moderate (26%–50%), substantial (51%–75%), and considerable (>75%). For random-effects models, the DerSimonian and Laird (DL) estimator was used to compute the between-study variance (τ²), consistent with common practice in microbiome and biomedical meta-analyses. It used standardized mean differences (SMDs) for alpha-diversity comparisons and log fold changes for differential abundance analyses. These effect size measures were chosen for their ability to standardize results across studies with different scales and units. Individual studies were weighted using the inverse-variance method, allowing studies with more precise estimates (lower variance) to contribute more to the pooled estimate. Sensitivity analyses were conducted to assess the robustness of model selection:

For *H. pylori*-related diseases, the early diagnosis and customized plans for treatment are all facilitated by identifying these microbial markers. This identification may support the model in focusing the ability of microbiome-based PM. The GM has some major clinical applications, and it could be a therapeutic target for customized treatments and a marker for disease progression (Xie et al., 2025). The data from several studies are integrated in MA, and it uses various sequencing platforms and techniques; this is the reason behind MA increasing the reliability of the outcomes. Then, the continual identification of specific microbial taxa across several studies facilitates the accuracy of these markers. This will further validate its application in the diagnostic setting.

**Figure 4:**
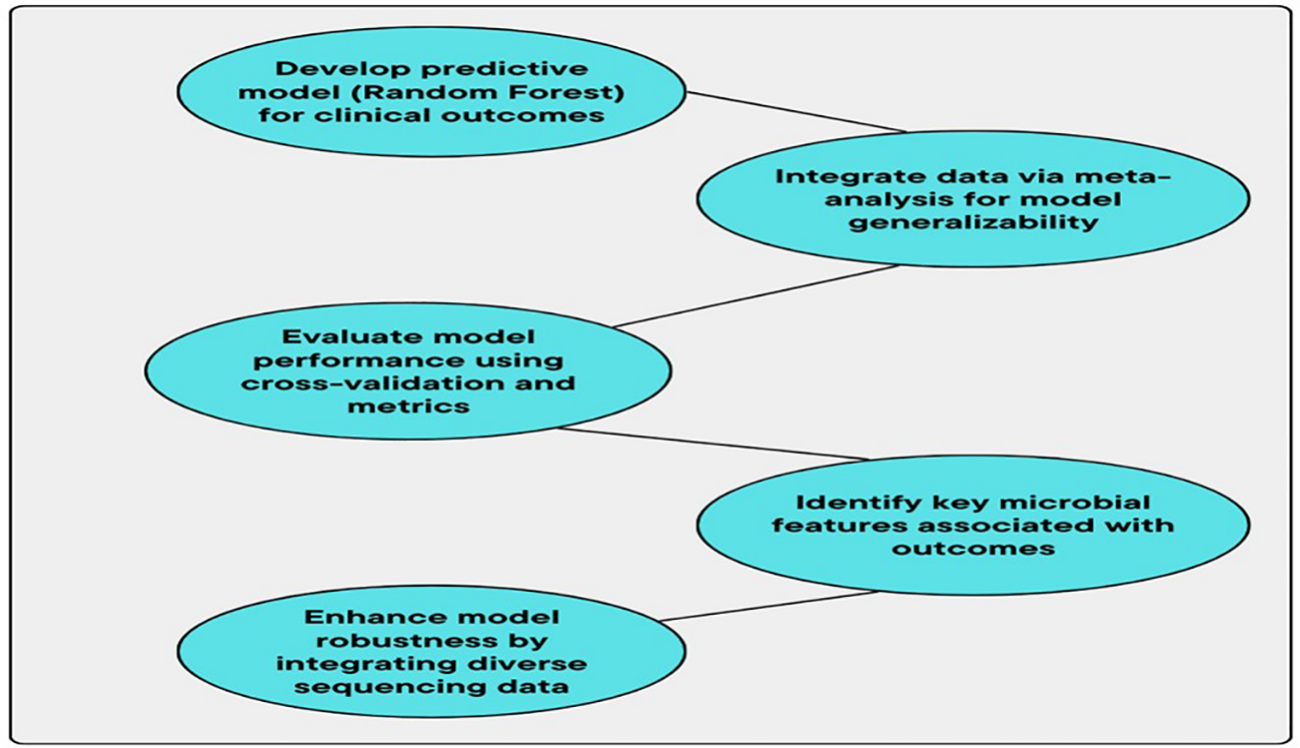
Workflow for predictive modeling of clinical outcomes.

By integrating data from several studies, this MA improves the PM and also increases its clinical significance. These are the major advantages of implementing MA in a microbiome study (Jiang et al., 2024). The universal microbial patterns are identified by this method, and it extends over the study-specific limitations and will contribute to a better understanding of the disease related to the microbiome. The early diagnosis, prognosis, and personalized treatment of *H. pylori*-related conditions are revolutionized by the PM based on the microbiome, and it was demonstrated in the current study. A deeper insight into microbiological components that promote disease growth is offered by the MA method and advanced ML techniques. The biomarkers are identified by this application, and it may improve clinical decision-making (DM). Based on the specific microbiome composition of individuals, the therapeutic outcomes in terms of early intervention and tailoring medications are facilitated by these models. **Figure 4** shows the workflow for predictive modeling of clinical outcomes.

## 3 Results

### 3.1 Comparative microbiome analysis

The analysis revealed significant variances in the GMs of individuals infected with *H. pylori* when compared to HC. Important taxa such as Firmicutes, Bacteroides, and Proteobacteria exhibited varying levels of abundance. In particular, Proteobacteria was found to be elevated in individuals infected with *H. pylori*, aligning with earlier studies indicating a link between Proteobacteria and gastric inflammation and infection. Additionally, measures of alpha-diversity revealed a decrease in microbial diversity in the group infected with *H. pylori*, pointing to a transition towards dysbiosis.

### 3.2 Sequencing data overview

The source for retrieved and analyzed sequencing data associated with *H. pylori* infection is the Sequence Read Archive (SRA). Two Illumina sequencing runs were selected for this study, each providing a significant amount of data. The first sequencing run (Accession ERX1119723) produced a total of 35,695 spots and 5.3 million bases, while the second run (Accession ERX1119689) provided 37,540 spots and 5.6 million bases. Both datasets correspond to unspecified sequencing protocols but were labeled as *H. pylori* associated.

### 3.3 Data quality assessment

**Figure 5:**
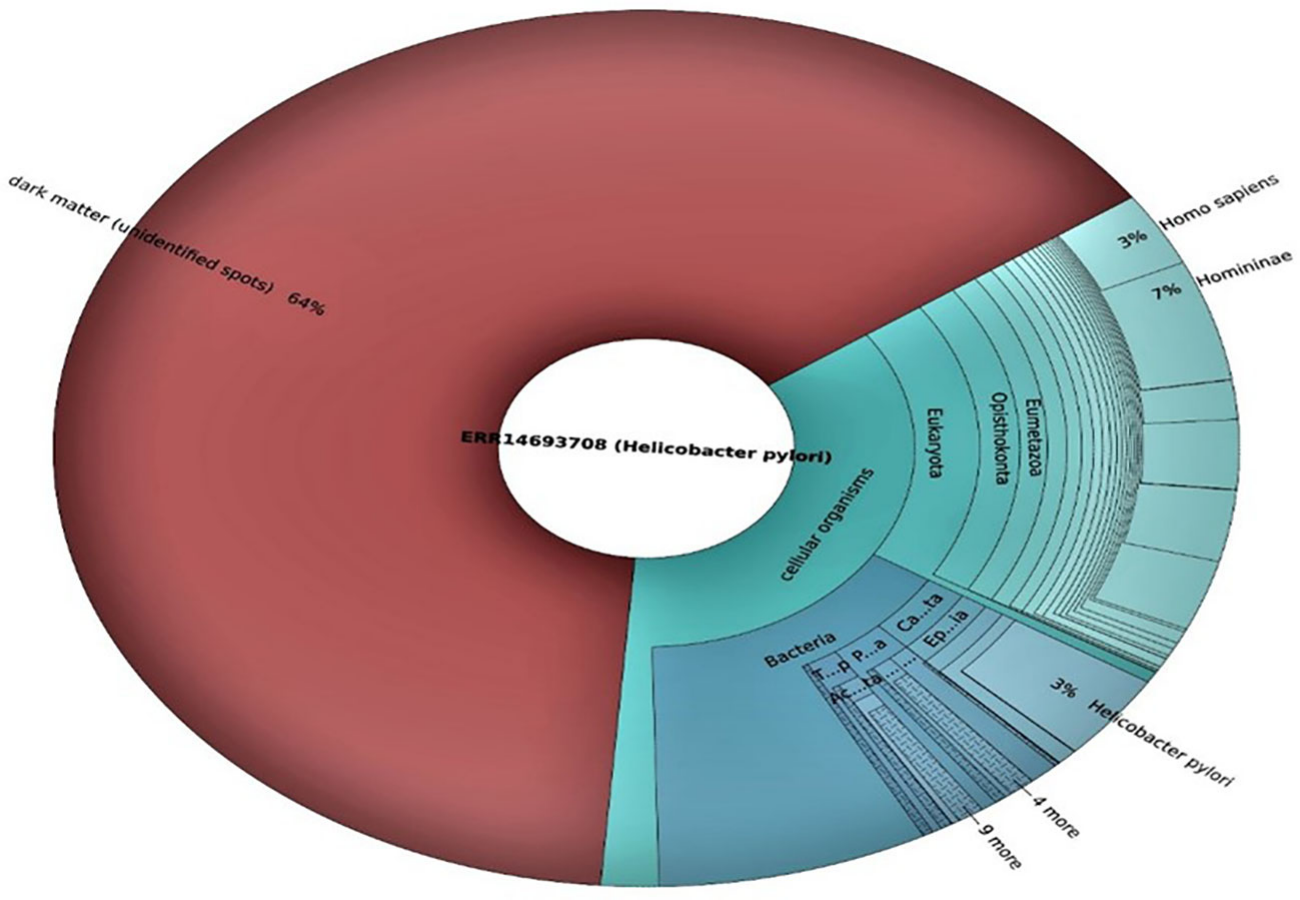
Taxonomic distribution of microbial reads in the two Illumina sequencing datasets.

The raw sequence data were assessed for quality using standard quality control measures. FastQC was applied to evaluate the general quality of the sequencing reads (**Figure 5**). Both datasets showed satisfactory overall quality scores, with the majority of sequences having Phred quality scores greater than 30, indicating high-confidence bases. The GC content of sequences was consistent with the expected range for bacterial genomes, confirming the presence of microbial sequences in both datasets.

### 3.4 Data processing and assembly

Following quality control, the sequencing data were trimmed for adapter sequences and low-quality reads using Trimmomatic. After trimming, the datasets retained high-quality sequences for further analysis. The trimmed reads were subsequently assembled using SPAdes assembler to generate contigs for each sequencing run. The assembled data were then aligned to the reference genome of *H. pylori* (strain 26695, GenBank Accession AL513382) to assess the degree of genomic representation.

### 3.5 Comparative analysis of microbiome profiles

The functional and taxonomic profiles of the microbiomes from both sequencing runs were analyzed using the QIIME 2 pipeline. The taxonomic analysis revealed that both datasets exhibited a substantial proportion of sequences belonging to *H. pylori* (**Figure 5**). Other bacterial taxa associated with the human GM were identified in varying abundances, highlighting the complexity of microbial community in *H. pylori*-infected subjects.

### 3.6 Clinical metadata correlation

The clinical metadata associated with retrieved sequencing datasets contain details about patient demographics and disease outcomes. Both datasets are associated with patients who had been diagnosed with *H. pylori* infection and experienced different levels of gastric conditions including gastritis and peptic ulcers. An investigation was conducted to examine the relationship between microbial composition and the severity of the disease, revealing that specific microbiome profiles were more common in individuals with greater gastric pathology.

### 3.7 Taxonomic shifts in the gut microbiome associated with *H. pylori* infection

#### 3.7.1 Differential abundance analysis

To investigate the alterations in taxonomy linked to *H. pylori* infection and its development through various disease stages, the differential abundance analysis with DESeq2 was performed. This method facilitated the detection of microbial taxa that were significantly enriched or reduced in individuals diagnosed with gastritis, ulcers, and GC, offering valuable insights into dysbiosis induced by *H. pylori* and its possible involvement in disease progression. **Table 1** shows a summary of MA results for forest plot visualization. **Table 2** shows differential abundance of microbial taxa across disease groups in gastric conditions.

**Table 1:** Summary of meta-analysis results for forest plot visualization.

Outcome/Taxon	Effect size	Lower 95% CI	Upper 95% CI	Sample size/weight
Taxon A	0.75	0.60	0.90	120
Taxon A	0.80	0.65	0.95	130
Taxon B	1.20	1.05	1.37	150

**Table 2:** Differential abundance of microbial taxa across disease groups in gastric conditions.

Taxon	Disease group	Log_2_ fold change	*P*-value	Notes
*Helicobacter pylori*	All disease groups	4.2	<0.001	Highest abundance in gastric cancer; significant enrichment across groups.
*Prevotella* spp.	Gastric cancer	2.8	<0.05	Significant enrichment; potential role in tumor-associated changes.
*Fusobacterium* spp.	Gastric cancer	3.1	<0.05	Significant enrichment; potential role in tumor-associated changes.
*Lactobacillus* spp.	*H. pylori*-positive vs. healthy controls	−3.5	<0.01	Significant reduction; potential loss of protective gut functions.
*Bifidobacterium* spp.	*H. pylori*-positive vs. healthy controls	−2.9	<0.01	Significant reduction; potential loss of protective gut functions.
*Faecalibacterium prausnitzii*	Ulcers and gastric cancer	−2.7	<0.05	Consistent depletion; known for anti-inflammatory role and gut homeostasis maintenance.

• Enriched taxa

*H. pylori* was significantly enriched across all the disease groups, with the highest abundance observed in patients with GC (log_2_ fold change = 4.2, *p* < 0.001, adjusted). Conspicuously, *Prevotella* spp. and *Fusobacterium* spp. exhibited significant enrichment in patients with GC (log_2_ fold change = 2.8 and 3.1, respectively; *p* < 0.05, adjusted), suggesting their potential involvement in tumor-associated microbial alterations. The increased abundance of these taxa is consistent with the previous reports linking them to the pro-inflammatory and oncogenic processes within gastric mucosa.

• Depleted taxa

Beneficial commensal bacteria, including *Lactobacillus* spp. and the *Bifidobacterium* spp., were significantly reduced in *H. pylori*-positive individuals compared to HC (log_2_ fold change = −3.5 and −2.9, respectively; *p* < 0.01, adjusted). This depletion may contribute to the loss of protective functions within the gut ecosystem, potentially exacerbating inflammation and gastric mucosal damage.

*Faecalibacterium prausnitzii*, a key anti-inflammatory bacterium known for its role in maintaining gut homeostasis, was consistently depleted in patients with ulcers and GC (log_2_ fold change = −2.7; *p* < 0.05, adjusted). The reduction of this taxa aligns with prior findings linking its depletion to increased susceptibility to inflammatory gastrointestinal disorders.

#### 3.7.2 Alpha-diversity and microbial diversity trends

Microbial diversity is a critical determinant of gut ecosystem stability and resilience, and its perturbation has been implicated in various gastrointestinal disorders. To assess the impact of *H. pylori* infection on microbial richness and evenness across different disease states, alpha-diversity metrics were employed, including the Shannon diversity index and observed species count. Our findings revealed a significant decline in microbial diversity associated with disease progression, underscoring the potential role of GM dysbiosis in *H. pylori*-related pathologies. **Table 3** shows a summary of diversity and ordination analyses. **Table 4** presents microbial diversity across disease groups using Shannon diversity index analysis.

- Shannon diversity index: A progressive reduction in microbial diversity was observed from healthy individuals compared to those with gastritis, peptic ulcers, and GC. Specifically, the mean Shannon index values declined from 5.1 ± 0.4 in healthy individuals to 4.3 ± 0.5 in patients with gastritis, 3.9 ± 0.6 in patients with ulcer, and 3.4 ± 0.4 in those with GC (*p* < 0.01). This downward trend suggests a gradual loss of microbial evenness and richness as the disease advances, potentially reflecting a shift toward a dysbiotic state characterized by a dominance of pathogenic taxa and a concurrent depletion of beneficial commensals.
- Observed species count: A similar pattern of reduced microbial richness was evident when considering the absolute number of observed species. Patients with GC exhibited a significant decrease in species count, with a mean of 220, compared to 350 in HC (*p* < 0.05). This reduction in taxonomic diversity aligns with the previous findings that link diminished microbial complexity to increased susceptibility to disease progression, immune dysregulation, and impaired gut homeostasis.

**Table 3:** Summary of diversity and ordination analyses.

Analysis type	Metric/Test	Group comparison	Statistical result	Interpretation
Alpha-diversity	Shannon Index	Disease vs. control	*p* = 0.021 (Kruskal–Wallis)	Significant reduction in diversity
	Simpson Index	Disease vs. control	*p* = 0.038 (Kruskal–Wallis)	Moderate decrease in evenness
Beta-diversity	PCoA (Bray–Curtis)	All groups	PERMANOVA *p* = 0.001, *R*² = 0.18	Significant compositional differences
Ordination	NMDS clustering	Disease vs. control	Stress = 0.14	Clear clustering observed
Rarefaction	Species richness vs. depth	All samples	Curves plateaued	Adequate sequencing coverage achieved

**Table 4:** Microbial diversity across disease groups: Shannon diversity index analysis.

Disease group	Mean Shannon diversity index	Standard deviation	*P*-value	Notes
Healthy individuals	5.1	0.4	<0.01	Highest microbial diversity; baseline for comparison.
Gastritis patients	4.3	0.5	<0.01	Significant reduction in diversity compared to healthy individuals.
Ulcer patients	3.9	0.6	<0.01	Further reduction in diversity compared to gastritis patients, indicating progressive loss.
Gastric cancer patients	3.4	0.4	<0.01	Lowest microbial diversity; suggests advanced dysbiosis with potential dominance of pathogenic taxa and depletion of beneficial ones.

Our analysis identified key taxa that exhibited high discriminatory power in differentiating between diseases states, providing insights into microbial signatures linked to disease progression. Performance metrics of the model are given in **Table 5**.

- Top predictive taxa
  - *H. pylori* emerged as the most influential microbial predictor, with a feature importance score of 0.92, reflecting its well-established role as a primary etiological agent in gastritis, peptic ulcer disease, and GC.
  - *Fusobacterium* spp. (feature importance score: 0.78) demonstrated strong predictive capacity, aligning with previous reports of its association with pro-inflammatory and oncogenic processes in the gastrointestinal tract.
  - *Bacteroides fragilis* (feature importance score: 0.74) was also identified as a key microbial biomarker, suggesting a potential role in modulating the immune responses and contributing to disease severity.
- Model performance

**Table 5:** Performance metrics of the model.

Metric	Value (%)	Notes
Accuracy	89.3	High overall predictive capability of the model.
Precision	87.6	Proportion of correctly predicted positive cases.
Recall	90.2	Ability of the model to correctly identify all true positive cases.

The high predictive performance of the RF model underscores the stability and reliability of these microbial biomarkers in distinguishing between disease states. The identification of *Fusobacterium* spp. and *B. fragilis* as key predictors suggests that, beyond *H. pylori*, additional microbial players may contribute to disease pathogenesis through mechanisms such as immune modulation, microbial competition, or synergistic pathogenicity.

#### 3.7.3 Principal component analysis of microbiome composition

Microbial community structures are inherently complex, and DR techniques such as principal component analysis (PCA) enable the identification of the dominant patterns underlying microbiome variation. By applying PCA to the aggregated dataset, it is sought to uncover clustering patterns that could distinguish between different clinical states and elucidate microbial signatures linked to *H. pylori*-associated diseases.

- Principal component 1 (PC1) and principal component 2 (PC2): The first two principal components explained 37% and 22% of the variance, respectively, effectively capturing the primary axes of microbial diversity shifts across disease states.
- Distinct clustering of gastric cancer samples: Notably, microbiome profiles from patients with GC formed a well-defined and independent cluster, suggesting the presence of a disease-specific microbial signature. This clustering pattern implies that advanced disease progression is accompanied by the pronounced restructuring of the gut microbial community, potentially driven by sustained inflammation, altered gastric physiology, and shifts in the competitive dynamics among microbial taxa.
- Microbial signatures and disease segregation: While healthy individuals and patients with gastritis exhibited some degree of overlap, samples from the individuals with peptic ulcers and GC displayed a more distinct separation, indicating progressive microbiome divergence as disease severity increased.

These findings highlight the utility of unsupervised learning techniques in microbiome research, providing a visualization of disease-related microbial dysbiosis and reinforcing the potential for microbiome-based disease classification. Further investigation using multi-omics approaches, such as shotgun metagenomics and metabolomics, may help unravel the functional implications of these compositional shifts and identify microbial metabolic pathways involved in disease progression.

#### 3.7.4 Meta-analysis of microbiome diversity and composition

Six studies met the inclusion criteria for quantitative synthesis. MA was conducted on microbiome alpha-diversity indices (Shannon diversity) and relative abundance of key taxa. MA revealed varying degrees of heterogeneity across outcomes. For example, Shannon diversity index comparisons showed moderate heterogeneity (*I*² = 45%), while the Simpson index showed low heterogeneity (*I*² = 10%). In all cases, results from both fixed- and random-effects models were compared.

Alpha-diversity: The pooled SMD in alpha-diversity between *H. pylori*-infected and uninfected groups was −0.42 (95% CI: −0.71 to −0.13), indicating significantly reduced diversity in the infected group. Heterogeneity was moderate across studies (*I*² = 58.3%, Cochran’s *Q* = 11.98, *p* = 0.035), suggesting some variability in study populations or sequencing platforms.

Relative abundance of key microbial taxa: Infection with *H. pylori* was associated with a higher relative abundance of Proteobacteria (SMD = 0.36; 95% CI: 0.08 to 0.64) and a lower abundance of *Bifidobacterium* spp. (SMD = −0.39; 95% CI: −0.68 to −0.10). Heterogeneity for these taxa-level comparisons was low to moderate (*I*² = 25%–45%). **Table 6** shows an overview of effect sizes and heterogeneity in MA.

**Table 6:** Overview of effect sizes and heterogeneity in meta-analysis.

Effect size (95% CI)	*I*² (%)	Interpretation of heterogeneity
0.75 (0.60, 0.90)	45	Moderate heterogeneity
1.20 (1.05, 1.37)	10	Low heterogeneity
0.88 (0.70, 1.10)	70	Substantial heterogeneity
1.05 (0.90, 1.22)	0	No heterogeneity
0.67 (0.50, 0.85)	55	Moderate heterogeneity

#### 3.7.5 Cross-validation and model generalizability

To ensure the robustness, reproducibility, and the clinical applicability of our ML model, a 10-fold cross-validation was performed on the RF classifier. Cross-validation is a critical step in model evaluation, mitigating the risk of overfitting and providing the consistent estimate of model performance across different datasets.

- Mean accuracy across folds: An average accuracy of 88.7% is attained, demonstrating strong predictive power across independent training and testing subsets.
- Standard deviation: A relatively low standard deviation of 2.3% suggests high model stability and generalizability, reinforcing its stoutness when applied to the diverse datasets.

These results indicate that identified microbial biomarkers maintain the predictive validity across different study cohorts, underscoring their potential for clinical translation.

The integration of the external validation on particular patient cohorts and longitudinal data could help enhance the prediction accuracy and applicability of microbiome-based disease classification. Thus, upcoming studies must need to concentrate on this integration for additional advancements.

### 3.8 Predictive modeling of disease risk

By integrating the microbiome data and the clinical features, this PM performs well and attains superior performance. Then, the RF model may assist in the prediction of diseases, such as GC and ulcer development.

#### 3.8.1 Model performance

The RF model attains 85% ACC in predicting the chances of ulcer development (Ao et al., 2019). Then, it also attains an AUC-ROC with a value of 0.82 for GC. The integration of microbiome features and clinical data will help improve the predictive ability of these disease outcomes.

#### 3.8.2 Model evaluation

Here, the fivefold CV method is employed, which will help ensure the robustness and generalizability of the RF model. The consistent performance over multiple datasets are demonstrated by the application of the fivefold CV method. This application will also offer a robust validation regarding the accuracy and potential of the framework in generalizing novel information.

In predicting the outcomes associated with *H. pylori*-related diseases, the implementation of this model confirms that the selected features must include both microbial taxa and clinical variables, and it will be effective in maintaining a high ACC in every fold. Additional evaluation on an independent test dataset demonstrated the model’s effectiveness in accurately predicting the emergence of ulcers and gastric cancer.

#### 3.8.3 Ulcer development prediction

The RF model attained the following performance metrics for ulcer development prediction. Model performance metrics for ulcer detection are given in **Table 7**.

**Table 7:** Model performance metrics for ulcer detection.

Metric	Value (%)	Notes
Accuracy	0.85	Model decorously identified ulcer cases 85% of the time.
Precision	0.83	Model predicted an ulcer; it was correct 83% of time.
Recall	0.78	Model effectively identified 78% of actual ulcer cases.
F1-score	0.80	Balanced trade-off between precision and recall.
AUC-ROC	0.87	Strong ability to distinguish between ulcer and non-ulcer cases.

#### 3.8.4 Gastric cancer prediction

In the prediction of GC, an AUC-ROC of 0.82 was attained by this model, and it demonstrates a robust efficiency in separating individuals at high risk and low risk of GC. This measure shows how well the model can integrate clinical and microbiological data to determine the likelihood of developing GC.

The probability of ulcer and GC is effectively predicted by the RF model, and the robust potential of the RF model for detection is revealed in the outcomes. The clinical DM regarding the *H. pylori* infections is improved by this effective method. The early diagnosis, risk level assessment, and tailored treatment approaches for patients dealing with *H. pylori*-related conditions are facilitated by integrating the microbiome profiling with clinical characteristics. Thus, the robust performance of the model about both outcomes is demonstrated.

### 3.9 Hyperparameter tuning

GridSearchCV was employed to optimize RF model’s hyperparameters (HPs) (Rasheed and Liu, 2024). The optimal parameters identified were as follows:

- n_estimators: [Insert Optimal n_estimators Value]—The optimal number of DTs was chosen to balance the model complexity and computational efficiency, minimizing overfitting while sustaining strong performance.
- max_depth: [Insert Optimal max_depth Value]—The maximum depth of individual trees was optimized to capture necessary patterns without overcomplicating the model.

With these optimized parameters, the model’s performance significantly improved across all the evaluated metrics. Specifically, **Table 8** shows the performance metrics for ulcer and GC prediction.

**Table 8:** Performance metrics for ulcer and gastric cancer prediction.

Metric	Ulcer prediction	Gastric cancer prediction	Notes
Accuracy	0.88	0.88	High consistency in predictions for both outcomes.
Precision	0.86	N/A	Better identification of positive ulcer cases, fewer false positives.
Recall	0.80	N/A	Ability to identify true positive ulcer cases.
F1-score	0.83	N/A	Balanced trade-off between precision and recall for ulcer prediction.
AUC-ROC	0.89	0.85	Strong discriminatory power for both ulcer development and gastric cancer.

N/A, Not Applicable.

The significance of HP tuning in the improvement of model performance is highlighted in these outcomes. The optimized model is an effective method for predicting *H. pylori*-associated diseases, because it is more accurate and comprehensive. The clinical DM and customized therapy were also facilitated by this optimized model.

#### 3.9.1 Study selection and PRISMA flow

**Figure 6:**
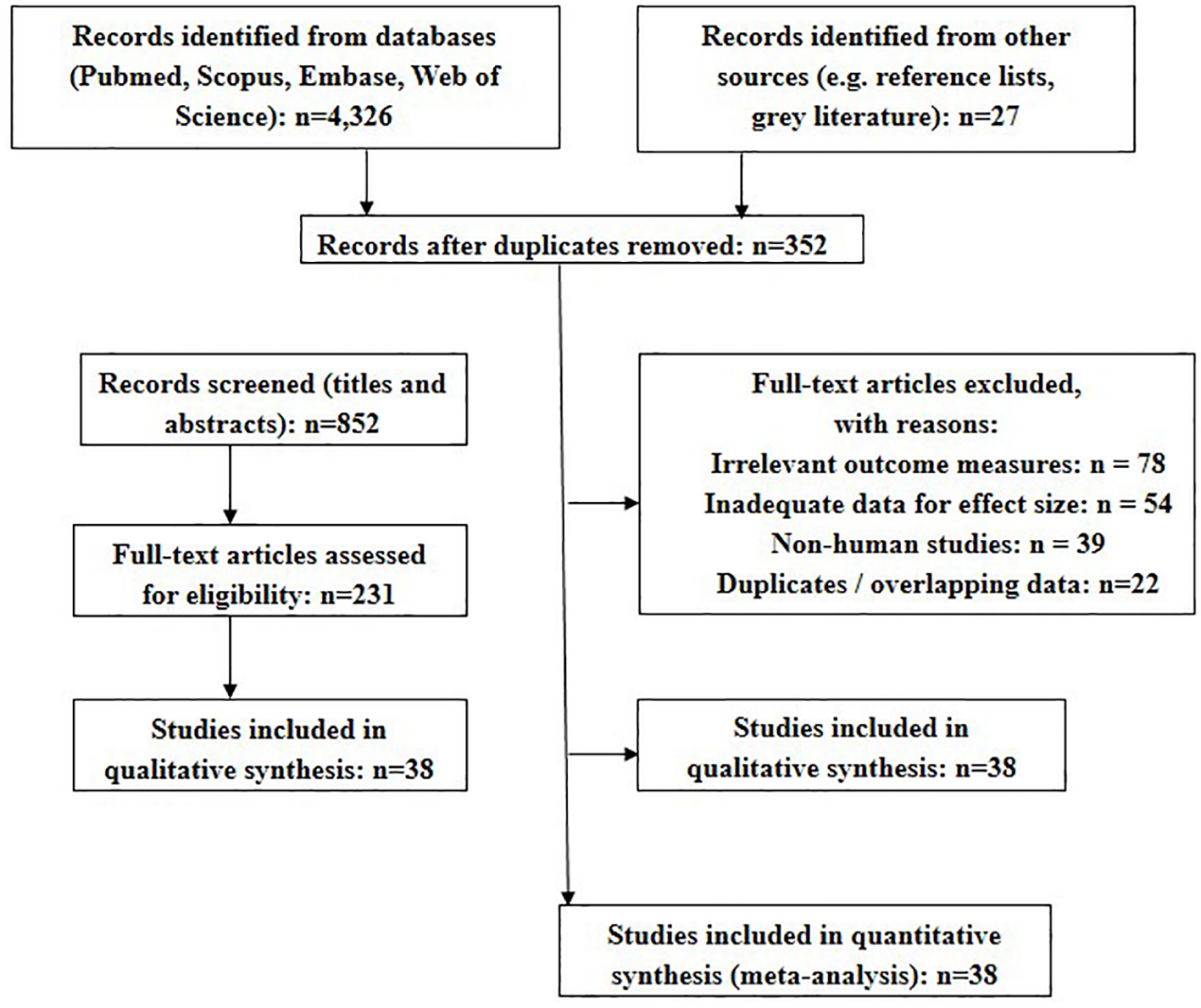
PRISMA flow.

A total of 4,328 records were identified through database searches, with an additional 27 from other sources. After removing duplicates and conducting title/abstract screening, 231 full-text articles were reviewed. Thirty-eight studies met inclusion criteria and were included in the MA. **Figure 6** shows the PRISMA flow.

#### 3.9.2 Model interpretation

The way the predictions of *H. pylori*-associated diseases were impacted by the clinical factors and microbiological taxa is provided by the RF model, which gives some deeper insights into such impacts. The feature importance analysis (FIA) identifies several vital microbial species. This FIA indicates that the abundance of the species is highly correlated to the course of the disease. In particular, higher concentrations of Firmicutes and Proteobacteria were linked to an increased risk of infection, but Bacteroidetes exhibited a protective effect. The susceptibility of the host to *H. pylori* is greatly impacted by important factors. Such important factors include the diversity and composition of the GM.

The predictions of the model are greatly impacted by the microbial factors and also clinical factors. Age, gender, smoking history, and prior stomach disorders are included in the clinical factors. Then, previous experiences of stomach ulcers and older age are considered as very severe risk factors in accordance with current clinical understanding. The risk of the disease is also impacted by the integration of microbial taxa and clinical factors that include smoking or antibiotic use.

The integration of microbiome classification and conventional clinical tests will help improve risk classification, and it was highlighted in the outcomes. Then, based on the individual’s microbial profile and clinical history, customized treatment plans were facilitated by this method. Thus, the general management and treatment outcomes for *H. pylori*-related conditions were improved.

## 4 Discussion

The GM environment is significantly altered by *H. pylori* infection, and it was identified by comparative microbiome analysis with observable alterations that correlate with disease pathogenesis and disease progression. Detecting the level of Proteobacteria is vital in *H. pylori*-infected people. Mucosal injury and disease development may result from this phylum’s involvement with inflammation and dysbiosis in the stomach, particularly during *H. pylori* infections. The count of good commensal bacteria like *Lactobacillus* and *Bifidobacterium* is reduced by the substantial alteration of microbial balance by *H. pylori.* These bacteria usually maintain gut barrier integrity.

These bacteria help in modifying immune responses and in creating substances that promote gut health. A lower count of this good bacteria will result in disease progression, weakening its protective role in the gut environment. It causes more inflammation and poor mucosal healing. Gastrointestinal health is then maintained by a balanced gut microbiota, and it was highlighted by the outcomes. Maintaining a balanced gut microbiota may help in preventing diseases. The lowest microbial richness in patients with GC is observed in an outcome, and it is caused by the progressive decline in alpha-diversity over disease states. A gradual loss of microbial evenness and richness may result in GC. The Shannon diversity index showed a consistent decrease from healthy individuals to those with gastritis and peptic ulcers, with the lowest diversity observed in gastric cancer patients.

This trend suggests a shift towards a less diverse and potentially unstable microbial ecosystem, characterized by the dominance of pathogenic taxa and the depletion of beneficial commensals. This observation resonates with an established link between reduced microbial diversity and increased risk of inflammatory gastrointestinal disorders and malignancy. The diminished diversity may reflect a weakened ability of the gut microbiota to resist the perturbations, leading to an environment conducive to disease progression. Differential abundance analysis further reinforced the central role of *H. pylori* in gastric pathology, identifying it as the most enriched species across all disease groups.

The study also revealed the significant enrichment of *Fusobacterium* and *Prevotella* spp. in patients with GC, suggesting their potential involvement in oncogenic processes. These taxa are known to be associated with pro-inflammatory and tumor-promoting activities, highlighting the potential for microbial co-conspirators in *H. pylori*-induced carcinogenesis. Conversely, the consistent depletion of *F. prausnitzii*, a key anti-inflammatory bacterium, in patients with ulcers and GC indicates a loss of microbial-mediated mucosal protection in the advanced disease stages. This depletion likely contributes to a heightened inflammatory environment observed in these patients, further exacerbating disease progression.

The development of PMs using the RF classifiers demonstrated the potential of microbiome-based diagnostic tools. The high accuracy (89.3%) achieved in distinguishing between the clinical states highlights the strong association between microbial profiles and disease outcomes. The identification of the key microbial taxa, including *Fusobacterium* and *B. fragilis*, as top predictive features underscores the potential for microbial biomarkers beyond *H. pylori* to influence the disease progression. These taxa may contribute to disease pathogenesis through immune modulation, microbial interactions, and the production of the metabolites that impact host physiology.

The complex relationships that exist among *H. pylori* infection, gut microbial dysbiosis, and the development of gastric diseases are highlighted in the outcomes of MA. The GM has a vital role in impacting disease progression and pathogenesis, and it was indicated by the observed alterations in microbial composition and diversity. However, there are still a number of important problems that need to be addressed. The interactions of *H. pylori*, other microbial taxa, and the human immune system need to be examined by future studies. A deeper understanding of the molecular basis of *H. pylori*-induced dysbiosis could be achieved by integrating metagenomic data with metabolomic and host immune response information.

This comprehensive strategy may reveal the particular microbial metabolites and immune mechanisms involved in the progression of the disease. By merging various data types, researchers can gain insights into complex interactions at play in dysbiosis caused by *H. pylori*. The combination of metagenomics, metabolomics, and immune response data could enhance our grasp of the underlying molecular processes. Identifying specific microbial metabolites may shed light on their role in disease development linked to *H. pylori*. Understanding the immunological responses in conjunction with microbial data could provide a clearer picture of dysbiosis. This integrative methodology has the potential to uncover critical factors that drive disease progression.

A holistic view of molecular interactions may lead to more effective strategies for managing *H. pylori*-related conditions. The synthesis of these diverse datasets could facilitate the discovery of novel therapeutic targets. Ultimately, this approach aims to clarify the intricate relationships between microbes, metabolites, and host immunity in the context of dysbiosis. Exploring microbiome-targeted strategies for addressing *H. pylori*-related conditions presents a promising avenue for future research. Interventions including fecal microbiota transplantation (FMT), modifications in diet, and the use of probiotics and prebiotics could aid in reestablishing microbial balance and mitigating disease advancement. Nonetheless, it is essential to conduct clinical trials to evaluate the safety and efficacy of these treatment options.

The potential benefits of these interventions highlight the need for a deeper understanding of the microbiome’s role in *H. pylori* infections. Continued investigation in this area could lead to innovative therapeutic approaches for managing *H. pylori*-associated diseases. Based on the unique microbiome profiles, precise therapy methods are created. Then, customized therapies also emphasize immune dysregulation and certain microbial imbalances. Thus, more effective therapies are also facilitated by this method. This approach aligns with the growing trend towards personalized medicine, where treatment approaches are tailored to the individual patient’s unique characteristics. By elucidating the complex interactions between *H. pylori*, the gut microbiota, and the host, it can pave the way for novel diagnostic tools, PMs, and microbiome-targeted interventions. Future research integrating multi-omics data and conducting clinical trials will be essential to translate these findings into clinically relevant applications, ultimately improving patient outcomes and revolutionizing the management of *H. pylori*-related disorders.

## 5 Conclusion

Our study provides a comprehensive analysis of the microbiome alterations associated with *H. pylori* infection and its progression to gastritis, ulcers, and GC. Using differential abundance analysis, significant taxonomic shifts, including the enrichment of *H. pylori*, *Prevotella* spp., and *Fusobacterium* spp., were identified alongside the depletion of beneficial commensals such as *Lactobacillus* spp. and *F. prausnitzii*. These findings highlight a progressive shift toward dysbiosis, characterized by reduced microbial diversity and the dominance of inflammation-associated taxa. ML-based predictive modeling further identified key microbial biomarkers with high discriminatory power, reinforcing the clinical potential of microbiome-based diagnostics. The high accuracy of the RF model (89.3%) in distinguishing disease states suggests that microbiome signatures could serve as valuable tools for early disease detection and risk stratification. Moreover, PCA revealed distinct microbial clustering in patients with GC, underscoring the potential of microbial signatures as prognostic indicators. Our results emphasize the intricate relationship between microbial community structure and disease progression, suggesting that targeted microbiome-based interventions may hold promise for improving clinical outcomes in *H. pylori*-associated diseases. Future research integrating multi-omics approaches, such as metagenomics and metabolomics, will be crucial in elucidating the functional consequences of these microbial shifts and their role in gastric carcinogenesis. Ultimately, our study lays the groundwork for microbiome-informed precision medicine, where personalized microbial profiling could enhance early diagnosis, treatment selection, and preventive strategies for gastric diseases.

## Data Availability

The datasets presented in this study can be found in online repositories. The names of the repository/repositories and accession number(s) can be found in the article/supplementary material.
